# 恶性胸腺瘤侵犯上腔静脉的外科治疗

**DOI:** 10.3779/j.issn.1009-3419.2018.04.05

**Published:** 2018-04-20

**Authors:** 林 许

**Affiliations:** 210009 南京，江苏省肿瘤医院 Jiangsu Cancer Hospital, Nanjing 210009, China

**Keywords:** 胸腺瘤, 上腔静脉, 外科治疗, Thymoma, Superior vena cava, Surgical treatment

## Abstract

恶性胸腺瘤侵犯上腔静脉在临床上较常见，但其外科治疗相关总结经验较少。本文详细介绍了恶性胸腺瘤侵犯上腔静脉外科治疗的发展历史与治疗现状，结合典型病例对恶性胸腺瘤合并上腔静脉综合征的影像学表征进行了探讨，重点描述了以外科手术为主的恶性胸腺瘤侵犯上腔静脉的综合治疗方法。该手术复杂程度高，手术风险大，为便于广大年轻医生的学习了解与实践操作，作者系统介绍了恶性胸腺瘤联合上腔静脉切除的4种手术方法，希望对年轻医生的学习应用有所裨益。

## Abstract (en)

This paper introduced surgical treatment of malignancy-related superior vena cava syndrome.Typical cases were presented with diagnostic radiology results.Authors focused on the main approach to the malignancy-related superior vena cava syndrome of surgery.In order to make it simple for junior doctors to learn and practice, all 4 operation methods were described in details.The writer hopes it would be helpful for all the young thoracic surgeons.

## 1 概述

胸腺瘤是前纵隔最常见的肿瘤，占所有纵隔肿瘤的20%-25%和前纵隔肿瘤的50%^[1]^。胸腺瘤是一种罕见的肿瘤，其生长模式通常非常缓慢。然而，它仍具有恶性潜质，因为它具有侵入局部组织和区域性转移的能力^[2]^。Masaoka分期是评估胸腺瘤分期最常用的分期系统^[3]^。Ⅲ期恶性胸腺瘤侵犯上腔静脉在临床上较常见。上腔静脉受侵最常见的体征为上腔静脉综合征(superior vena cava syndrome, SVCS)，是由于恶性胸腺瘤压迫或侵犯上腔静脉引起的完全性或不完全性上腔静脉阻塞，导致上腔静脉系统血液回流受阻，出现上肢、颈和颜面部发绀、水肿，以及上半身表浅静脉曲张的一组临床综合征^[4]^。恶性胸腺瘤一旦合并SVCS，传统的治疗方法是脱水、激素、上腔静脉内支架、放疗和化疗治疗。虽然可暂时部分缓解上腔静脉梗阻，但疗效极差，绝大多数患者在3个月-6个月内死亡。在20世纪60年代中后期国内外部分学者曾应用大隐静脉——颈外静脉吻合术治疗上腔静脉梗阻，但近远期疗效均不佳，绝大多数患者均在短期内死亡。80年代末和90年代初，国内外学者开始尝试应用血管内支架加化放疗治疗恶性胸腺瘤合并SVCS，使部分患者获得较好的近期疗效，但无长期生存病例。90年代以后，国内外相继有学者^[5]^报道恶性胸腺瘤联合上腔静脉切除、人造血管置换术治疗恶性胸腺瘤伴SVCS获得良好的近期和远期效果。本科室对恶性胸腺瘤伴SVCS施行上腔静脉切除、人造血管重建术，5年生存率为28.56%，生存时间最长的已超过13年。外科治疗能明显改善恶性胸腺瘤伴SVCS的近期和远期效果，对恶性胸腺瘤伴侵犯上腔静脉的病例，不应轻易放弃手术。

## 2 诊断

恶性胸腺瘤合并上腔静脉综合征的诊断，主要靠临床症状、胸部计算机断层扫描(computed tomography, CT)、胸部磁共振成像(magnetic resonance imaging, MRI)检查以及正电子发射计算机断层显像(positron emission tomography/computed tomography, PET/CT)检查^[6]^。

### 2.1 临床症状

恶性胸腺瘤合并上腔静脉综合征通常出现上腔静脉头面、上肢水肿；胸部静脉曲张。

### 2.2 胸部CT

扫描CT和MRI可以明确显示恶性胸腺瘤侵犯上腔静脉的部位、范围及类型，是外科医师决定手术指征和手术方式的主要依据。

#### 2.2.1 胸部CT

CT诊断上腔静脉或其主要分支阻塞至少需要两个征象：①阻塞部位以远的中心静脉无显影(或显影淡)；②侧支静脉血管显影。增强扫描可清楚显示不同的血管管腔，精确诊断腔静脉受压的部位、程度和可能原因、侧支循环通路、静脉扩张程度，便于决定治疗方案。

#### 2.2.2 MRI

MRI具有X、Y、Z轴三个方向上的梯度磁场，故能进行多方位扫描成像，避免了CT只能横断扫描的不足。并且因其流空效应，不用造影剂的情况下，MRI即能对胸廓入口及纵隔大血管清楚显示。同时亦能精确地给出血管阻塞的部位、范围及可能的原因。

## 3 手术适应证

侵犯上腔静脉的胸腺瘤均为Ⅲ期恶性胸腺瘤，保守治疗预后极差，手术为主的综合治疗可直接切除病灶，合理辅以放或化疗，可明显提高相当一部分患者的生存率和生活质量，但应掌握好手术适应症适应症，其适应症包括：患者一般情况良好，各脏器功能基本正常能耐受本手术者；经临床检查、CT或MRI及全身同位素骨扫描，确定肿瘤局限，而无远处转移者；可经手术完全切除者。

## 4 手术治疗

### 4.1 恶性胸腺瘤联合上腔静脉切除的4种方法(图 1-图 5)

**1:**
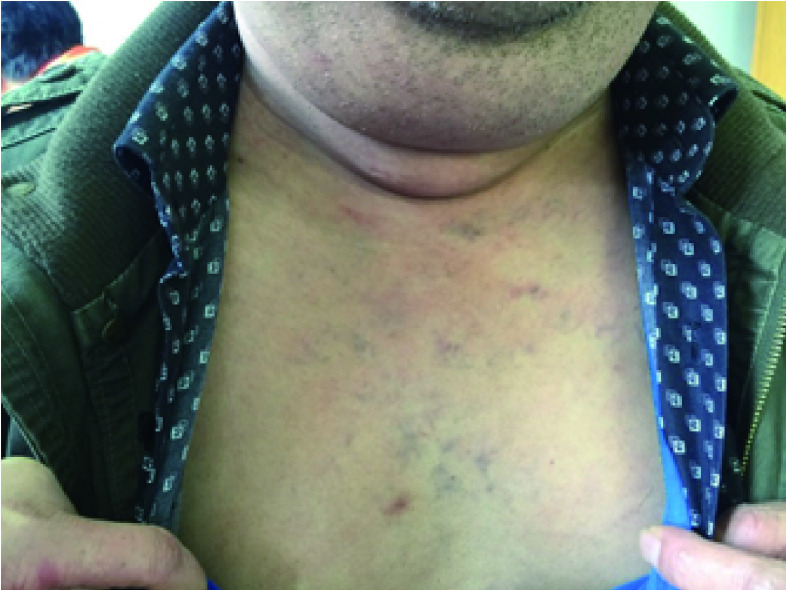
恶性胸腺瘤合并上腔静脉综合征 Malignancy-related superior vena cava syndrome

**2:**
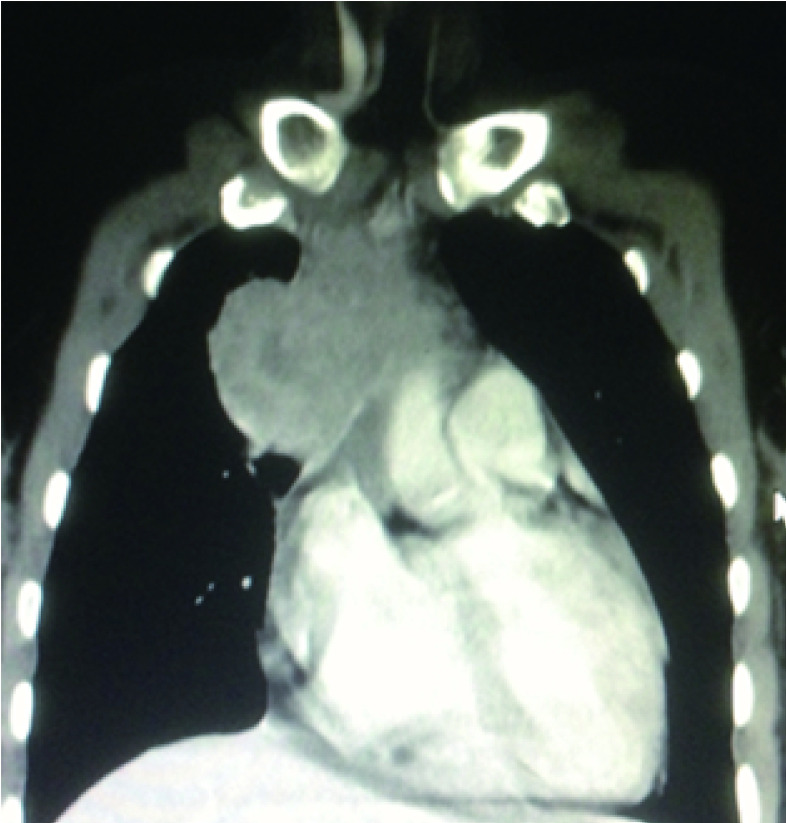
恶性胸腺瘤侵犯上腔静脉的CT征象 CT images malignancy-related superior vena cava syndrome.CT: computed tomography.

**3:**
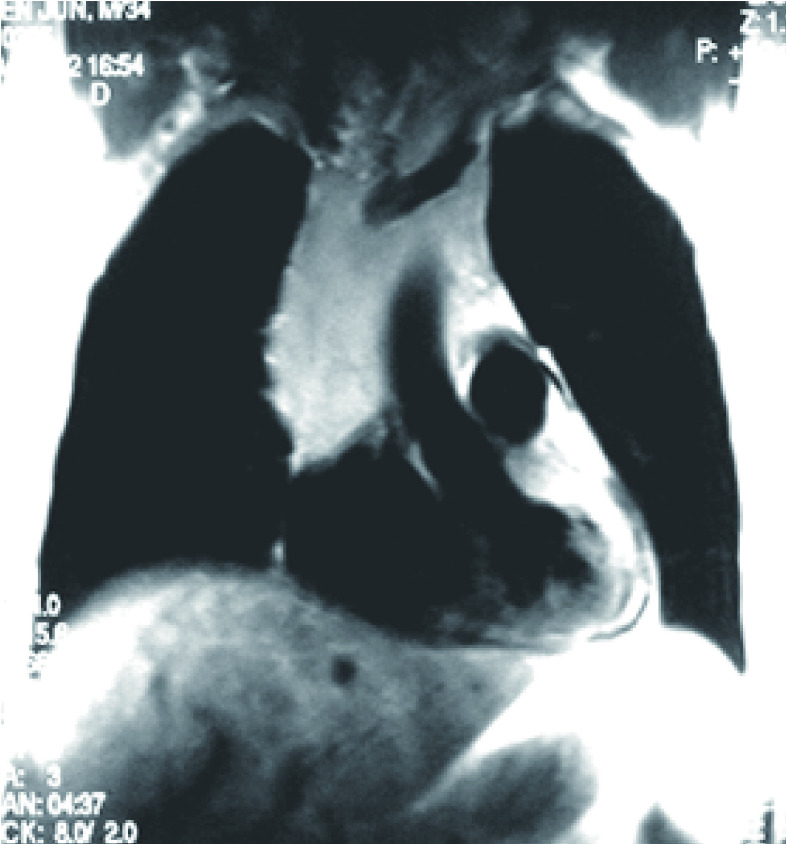
恶性胸腺瘤侵犯上腔静脉的MRI征象 MRI images of malignancy-related superior vena cava syndrome.MRI: magnetic resonance imaging.

**4:**
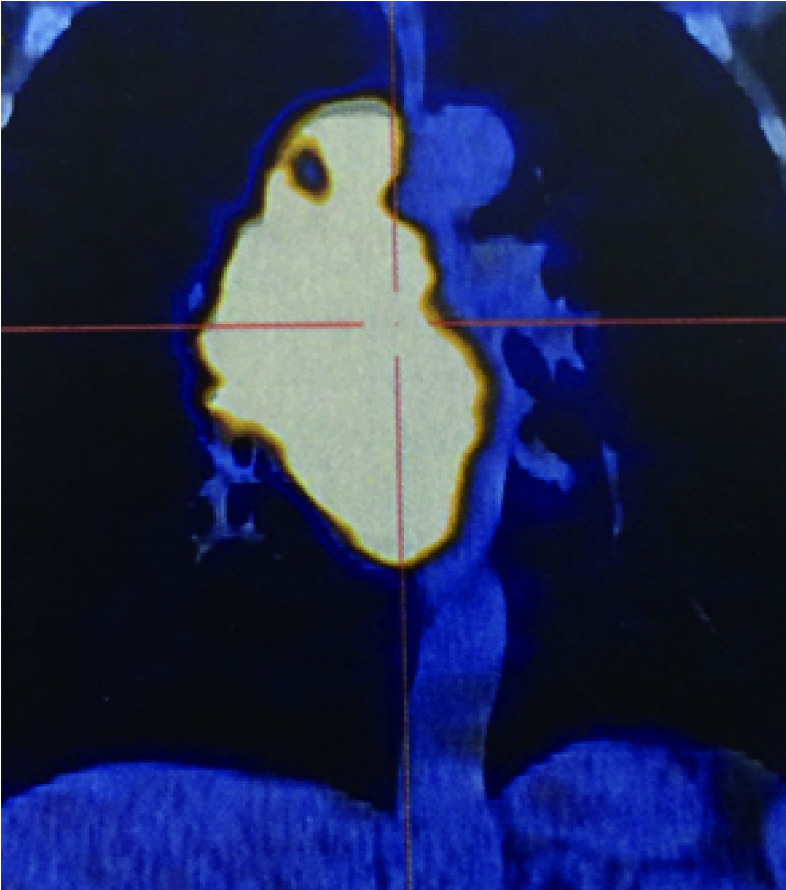
恶性胸腺瘤侵犯上腔静脉的PET/CT征象 PET/CT images of malignancy-related superior vena cava syndrome.PET/CT: positron emission tomography/computed tomography.

**5:**
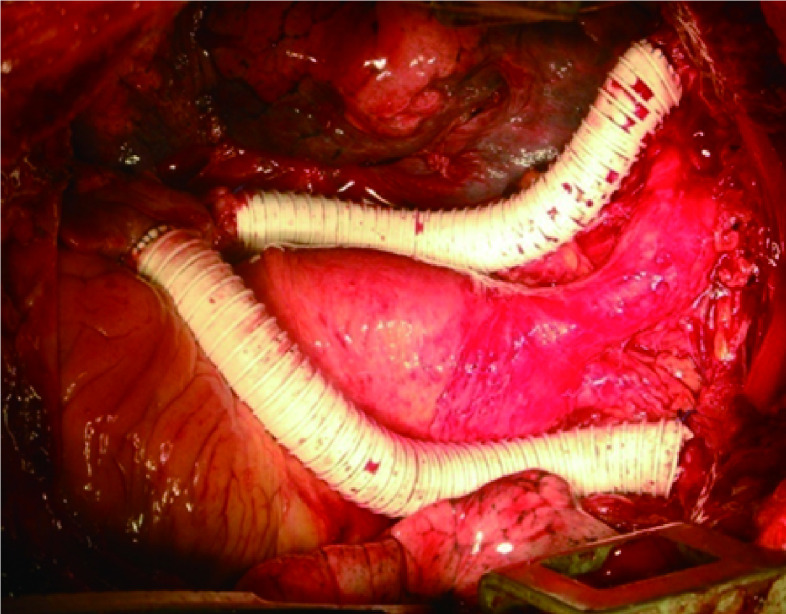
不阻断上腔静脉血液回流的人工上腔静脉置换 Artificial superior vena cava replacement without blocking the reflux of superior vena cava

(1) 完全阻断SVC后切除病变与上腔静脉，再以人工血管替代手术步骤如下：①常规气管内插管，静脉麻醉。②胸骨正中纵劈切口或加用前外侧切口，探查恶性胸腺瘤侵犯上腔静脉及其它器官情况。③切开心包解剖左、右无名静脉，心包内段上腔静脉，放置阻断线。④解剖奇静脉，分别在近、远端结扎，缝扎，然后切断奇静脉弓。⑤用无损伤血管阻断钳分别阻断左右无名静脉和心包内段上腔静脉，持续监测右无名静脉内压力，如压力较阻断前上升超过20 mmHg，则经右颈部的静脉血管鞘导管放血经含抗凝剂的输血器经下肢静脉通道回输。⑥在左右无名静脉汇合处下方和上腔静脉与右心房汇合处上方联合切除恶性胸腺瘤侵犯的上腔静脉。用肝素生理盐水冲洗上腔静脉近、远端血管腔，选择恰当的Cortex人造血管行上腔静脉重建。⑦用4-0的Prolene线，将人造血管与上腔静脉远端行端端连续缝合。将人造血管近心端与心包内段上腔静脉行端端吻合，或人造血管近心端与右心房或右心耳端侧吻合。⑧在吻合最后2针前，先开放左右无名静脉阻断钳，排除人造血管内的空气，待近心端人造血管吻合完成后，开放近心端上腔静脉阻断钳，或右心房壁上的心耳钳^[7]^。⑨抗癌药生理盐水冲洗心包腔和胸膜腔。(2)不阻断SVC，在左(或)右无名静脉与右心耳(或右心房)之间用人工血管搭桥后，切除病变与上腔静脉手术步骤如下：①-④同4.1.1；⑤切断左(或)右无名静脉用人工血管与右心耳(或右心房)之间搭桥。⑥切断另一根无名静脉，在上腔静脉与右心房汇合处上方切断上腔静脉，连同病变一并切除上腔静脉。用肝素生理盐水冲洗上腔静脉近、远端血管腔，选择恰当的Cortex人造血管准备行另一根无名静脉与上腔静脉近心端吻合，重建上腔静脉。⑦用4-0的Prolene线，将人造血管与无名静脉行端端连续缝合。将人造血管近心端与心包内段上腔静脉行端端吻合，或人造血管近心端与右心房行端侧吻合。⑧-⑨同4.1.1。(3)不阻断SVC，应用上腔静脉内引流管保证静脉回流，切除病变及部分腔静脉壁①-③同4.1.1。④用4-0的Prolene线在右心房上荷包缝合，切开荷包线内的右心房。将1根20 F近端有侧孔之硅胶管经右心房向右无名静脉内插入，并用阻断带阻断病变远近端的上腔静脉及分支，保证静脉回流。⑤切除腔静脉壁上的病变并修剪与上腔静脉缺损相同大小的心包片或其他材料加以修补，重建上腔静脉。⑥在修补完毕后，拔除上腔静脉内引流管，收紧右心房上的荷包线打结，将右心房切口修复，去除所有血管阻断带。⑦抗癌药生理盐水冲洗心包腔和胸膜腔。(4)不阻断SVC，用侧壁钳钳夹住腔静脉一侧壁，切除静脉壁上的病变并加以修补手术步骤如下：①-②同4.1.1。③切开心包充分解剖上腔静脉全长，放置阻断带预防上腔静脉出血。④用侧壁血管钳夹住腔静脉一侧壁(不超过上腔静脉直径的1/2)，不影响静脉血回流，切除静脉壁上的病变直接缝合或用其他材料加以修补。⑤在静脉壁缝合或修补完毕后，放松侧壁血管钳。⑥抗癌药生理盐水冲洗心包腔和胸膜腔。

### 4.2 手术要点

(1) 力争完整切除受侵的SVC和肿瘤：恶性胸腺瘤易直接侵犯上腔静脉，应采取将受侵的上腔静脉连同肿瘤组织一并完整切除的方法，尽量不用分块切除方法减少和避免肿瘤细胞脱落在胸腔内种植的机会。(2)重视脑保护：①控制上腔静脉阻断时间：完全阻断上腔静脉时间，应控制在30 min内，一般不会出现神经损害等严重并发症，②在上腔静脉阻断前；采用控制性降低血压、冰帽降温等。③在上腔静脉阻断中：尽可能缩短上腔静脉阻断时间，先完成人工血管与右心房的吻合后再阻断上腔静脉，切除肿瘤，然后行人工血管与上腔静脉远端的对端吻合。阻断时间一般在20 min内，脑损伤大大减轻。(3)不阻断上腔静脉血液回流：在切除上腔静脉前，先实行右心房与无名血管人工血管搭桥，使上腔静脉血液回流不受影响，然后手术切除及吻合，手术安全而从容，作者常采用此种方法。(4)术中术后防止血栓形成：①上腔静脉阻断前应用静脉肝素化，并用肝素盐水浸泡人工血管可防止操作时其内形成血栓。②采用外翻缝合吻合上腔静脉和人造血管，使吻合口内壁光滑。③开放上腔静脉阻断时应先开放远心端，使人造血管充盈，排气后再开放近心端，防止血循环内气栓。④切断奇静脉减少侧支循环，增加中心静脉血量从而减少人造血管内血栓形成的机会。⑤术后第2天起用肝素和华法林抗凝，减少人造血管内血栓形成。(5)注意人造血管的选择：可选用Gore-Tex或国产涤纶人造血管，但前者组织相容性及缝合严密性较好而且带有环，不会因为受挤压等造成管腔狭窄，应为首选。人造血管直径较上腔静脉直径小或大1 mm-2 mm均可顺利地进行吻合，一般为12 mm-18 mm。

## 5 术后并发症

### 5.1 术后吻合口出血

胸腔引流量多，诊断为吻合口出血时，应及时开胸止血，缝合吻合口出血部位。

### 5.2 术后血管栓塞

术后发生上腔静脉综合症的患者，首先应考虑为血管栓塞，应行增强胸部CT、心脏彩超检查，诊断为置换之血管栓塞者，应及时开胸切除人造血管、取出血栓后再行吻合，国内有这样的病例报道。但亦有报道术后发生上腔静脉血栓未手术，而采取全身肝素化治愈。

## 6 总结

恶性胸腺瘤合并SVCS行病灶加全上腔静脉切除、人造血管置换术的手术结果，近年国内外均仅有少量病例报道^[8-10]^。由于手术操作困难、风险大、对术者手术技巧要求很高。现有的结果表明术后上腔静脉梗阻症状可在短期内消失，人造血管通畅，相当一部分患者可获长期生存。国内外胸外科专家认为采用人工血管置换受侵的上腔静脉提高了恶性胸腺瘤的手术切除率，无严重致命并发症的发生并获得了较高的术后生存率，值得在恶性胸腺瘤的外科治疗中得以推荐应用。
